# Evaluating the Clinical Impact of an Enhanced Recovery After Surgery (ERAS) Program: A Quality Improvement and Practice Change Study at Al-hada Armed Forces Hospital

**DOI:** 10.7759/cureus.117194

**Published:** 2026-09-30

**Authors:** Alaa M Abdelhafeez, Hussain O Alshehri, Samah S Nooh, Khalid Al Towairgi, Budur T althobaiti, Abdulaziz Alotaibi, Mohammed S Banjar, Muhannad A Alshehri, Thirusha Govender, Mohammed o Gadour

**Affiliations:** 1 Anesthesia, Al-Hada Armed Forces Hospital, Taif, SAU; 2 Anesthesia, Omdurman Islamic University, Khartoum, SDN; 3 General Surgery, Al-Hada Armed Forces Hospital, Taif, SAU; 4 Quality Improvement, Al-Hada Armed Forces Hospital, Taif, SAU; 5 Internal Medicine, Omdurman Islamic University, Khartoum, SDN

**Keywords:** cost-effectiveness analysis, ​enhanced recovery after surgery, financial impact, laparoscopic cholecystectomy, patient satisfaction score, ​perioperative care, quality improvement projects, reduce length of stay

## Abstract

Introduction

Enhanced recovery after surgery (ERAS) is a proven perioperative framework designed to attenuate surgical stress and accelerate recovery. However, translating global ERAS evidence into localized practice requires bridging significant implementation gaps and modifying entrenched clinical habits. This study evaluates the clinical and operational impact of a multidisciplinary ERAS implementation model for laparoscopic cholecystectomy at a tertiary military hospital.

​Methods

This quasi-experimental pre- and post-intervention quality improvement study at Alhada Armed Forces Hospital evaluated an ERAS protocol for elective laparoscopic cholecystectomy (aged 18-80 years, ASA I-III), comparing prospective post-intervention (n = 120) and retrospective baseline (n = 88) cohorts. Excluded patients included emergency cases, severe comorbidities, cognitive impairment, or open conversions (30 excluded; 208 analyzed). Interventions spanned three plan-do-study-act (PDSA) cycles featuring opioid-reducing analgesia, standardized perioperative care, point-of-care checklists, and protocol-driven discharge. Clinical, economic, and satisfaction outcomes were extracted from hospital databases and SMS surveys, then analyzed in jamovi via Student's t-tests and Chi-square (χ2 ) tests (p < 0.05).

​Results

Among 208 analyzed patients (baseline: n = 88; ERAS: n = 120), ERAS implementation increased checklist compliance to 100.0% (mean: 72.1% ± 38.7%). Ambulatory day-case success increased from 0.0% to 27.5% (33/120; p < 0.0001), while mean length of stay (LOS) dropped from 4.33 ± 0.89 to 2.67 ± 1.15 days (t = 3.96, p = 0.0007). Implementation of the ERAS protocol saved 231 inpatient bed days across 77 patients, achieving total cost savings of Saudi Riyal (SAR) 346,500 [$92,400 United States Dollars (USD)]. Postoperative complications were recorded in only one patient (1/120; 0.83%), with zero surgical site infections (0.0%). Patient satisfaction averaged 89.4% ± 3.3%, with a 30-day readmission rate of 0.83% (1/120).

Conclusion

Integrating a file-embedded ERAS checklist into routine multidisciplinary care safely reduces LOS and expands same-day discharge for laparoscopic cholecystectomy without sacrificing safety, outcomes, or patient satisfaction. By maximizing existing infrastructure and structured patient education, this low-cost model provides a practical framework for healthcare systems seeking to optimize ambulatory capacity and operational efficiency.

## Introduction

As a paradigm-shifting multidisciplinary framework, enhanced recovery after surgery (ERAS) pathways dismantle traditional perioperative dogmas to attenuate surgical stress and preserve physiological homeostasis. By seamlessly integrating evidence-based preoperative, intraoperative, and postoperative interventions, ERAS significantly optimizes clinical throughput, accelerates recovery, and minimizes hospital stays [1]. These protocols offer a structured, evidence-based framework that revolutionizes surgical outcomes by optimizing patient physiology and minimizing surgical stress. By integrating key tenets-including abbreviated fasting with carbohydrate loading, multimodal opioid-sparing analgesia, goal-directed fluid therapy, and early mobilization-the ERAS philosophy successfully transforms the patient journey from a passive experience into an accelerated, proactive recovery [2,3].

Historically, major surgery was associated with significant patient burden during post-operative recovery, characterized by prolonged fasting, aggressive bowel preparation, routine nasogastric decompression, and mandatory bed rest. This standard approach shifted in the 1990s following Henrik Kehlet’s landmark research on the physiological stress response to surgical trauma. Kehlet identified this neuroendocrine and metabolic surge as the primary driver of post-operative organ dysfunction. He hypothesized that a coordinated, multimodal pathway could attenuate this response, ultimately reducing complication rates and establishing the framework for modern enhanced recovery protocols [4].

The integration of these standardized pathways has demonstrated remarkable efficacy in mitigating common barriers to timely discharge, such as uncontrolled surgical pain, urinary retention, and postoperative nausea and vomiting (PONV) [5]. Meta-analytic data consistently highlight that clinical standardization significantly compresses the post-operative length of stay (LOS) and drives successful day-case or ambulatory surgery conversions without escalating 30-day readmission rates or baseline surgical complications [6,7].

​Despite robust evidence validating the clinical benefits of ERAS pathways, global compliance remains highly variable, particularly within resource-constrained or culturally traditional settings. Widespread realization of day-case laparoscopic cholecystectomy (LC) outcomes is frequently impeded by entrenched surgical habits, disparate patient expectations, and inadequate interdepartmental communication. Overcoming these systemic barriers requires a cohesive multidisciplinary framework, strong organizational commitment, and real-time compliance audits to successfully transition from traditional practices to standardized, evidence-based care [8].

​​The primary objective of this study was to evaluate the clinical and economic impact of implementing a multidisciplinary ERAS pathway on mean LOS, day-case conversion rates, and healthcare costs for patients undergoing elective laparoscopic cholecystectomy. Secondary objectives were to assess its impact on 30-day readmission rates, postoperative complications, and overall clinical recovery.

## Materials and methods

Study design, setting, duration, and study population

This quasi-experimental, pre-and-post-intervention study was conducted at Alhada Armed Forces Hospital (Taif, Saudi Arabia) to evaluate the clinical and economic impacts of standardizing perioperative care via an ERAS protocol. The study evaluated a prospective post-intervention cohort (January 2025 to December 2025) managed under the ERAS pathway against a retrospective, historical baseline cohort (January 2024 to December 2024) managed under traditional care. Following formal institutional approval, eligible participants were elective laparoscopic cholecystectomy patients aged 18 to 80 years with an American Society of Anesthesiologists (ASA) physical status of I, II, or III [9]. Written informed consent was obtained for all prospectively enrolled patients. Exclusion criteria comprised emergency cases, severe decompensated comorbidities (specifically cardiopulmonary, cerebrovascular, renal, or hepatic diseases), cognitive impairments preventing compliance, or intraoperative conversion to open surgery. Out of 238 screened patients, 30 were excluded, leaving a final sample size of N = 208 patients for analysis (baseline cohort, n = 88; ERAS cohort, n = 120).

​Implementation strategy and plan-do-study-act (PDSA) cycles

This multidisciplinary ERAS project spanned 12 months (January-December 2025), incorporating an active eight-month clinical implementation phase (May-December 2025) across outpatient clinics, the day surgery unit, operating rooms, the postanesthesia care unit, and inpatient surgical wards. Overseen by an institutional steering committee of surgeons, anesthesiologists, specialized nurses, dietitians, and physiotherapists, perioperative care pathways were structured according to the guidelines of the ERAS Society [10]. Protocol adherence was prospectively monitored and audited at each point of care using a mandatory, study-specific ERAS checklist (Figure 1). 

**Figure 1:**
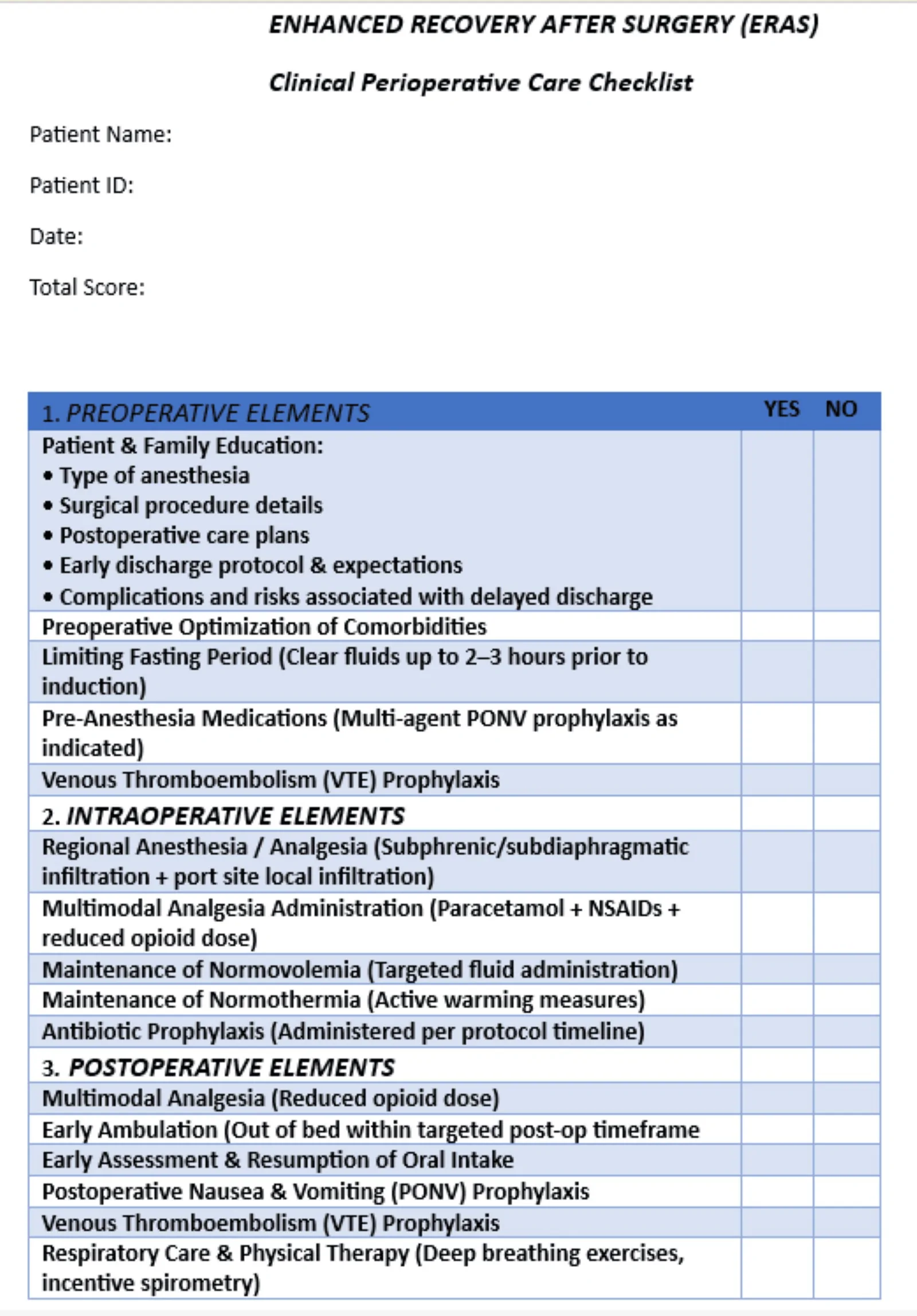
Clinical perioperative care checklist for ERAS ID: identification; PONV: postoperative nausea and vomiting; VTE: venous thromboembolism; NSAIDs: non-steroidal anti-inflammatory drugs; ERAS: enhanced recovery after surgery. *Checklist developed for clinical implementation based on established ERAS Society guidelines [10].

Tailored interventions included preoperative patient education, standardized general anesthesia induction (fentanyl 1-2 µg/kg, propofol 2 mg/kg, lidocaine 1 mg/kg, rocuronium 0.5 mg/kg), prophylactic cefazolin (1 g), non-opioid analgesics (paracetamol 1 g, lornoxicam 8 mg), antiemetics (dexamethasone 8 mg, ondansetron 8 mg), subphrenic 1% lidocaine instillation (10 mL), and port-site infiltration (5 mL 1% lidocaine with 5 mL 0.5% bupivacaine). Goal-directed intraoperative fluid management and active thermal management maintained normovolemia and normothermia throughout the perioperative period. Postoperative care emphasized post anesthesia care unit (PACU) rescue analgesia for pain scores ≥ 4 (morphine 0.05-0.1 mg/kg or pethidine 0.5-1 mg/kg), early oral intake, and early ambulation. Protocol implementation was refined across three PDSA cycles. In PDSA cycle 1, lecture-based staff education was conducted alongside preoperative patient education; however, initial audit data proved lecture-based sessions ineffective, resulting in poor baseline protocol compliance due to inconsistent adherence and a lack of point-of-care tools. To overcome this, PDSA cycle 2 replaced passive education by integrating a best practice advisory (BPA) checklist directly into clinical workflows, achieving high compliance through mandatory verification and dual sign-offs across perioperative units. Finally, PDSA cycle 3 optimized discharge logistics from both the ward and day surgery unit via electronic order sets, standardized discharge criteria, and 24-hour post-discharge telephone follow-ups targeting a one-day LOS (Figure 2). 

**Figure 2:**
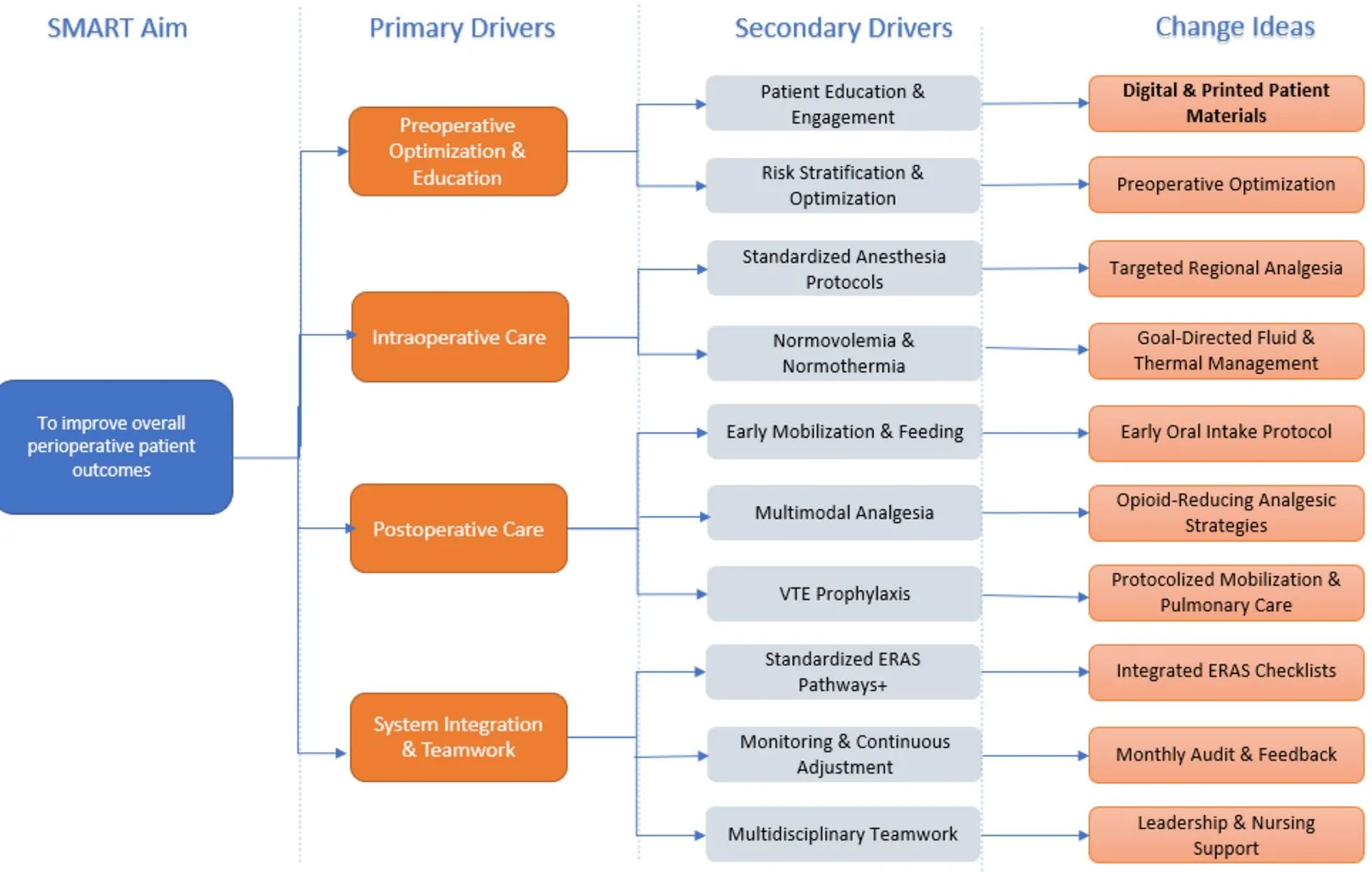
Driver diagram mapping targeted change ideas across the ERAS continuum SMART: specific, measurable, actionable, relevant, and time-bound; VTE: venous thromboembolism; ERAS: enhanced recovery after surgery.

The protocol successfully achieved safe postoperative day-one clinical discharge, despite hospital electronic systems recording a two-day administrative LOS due to automated midnight-occupancy algorithms.

Cost and resource analysis

Direct hospital estimated bed-day resource savings were calculated for May-December, corresponding to the post-implementation period of LOS reduction. Savings were based on total inpatient days saved between cohorts, valued at the official Ministry of Defense Health Services (MODHS) standard rate of Saudi Riyal (SAR) 1,500 [$400 United States Dollars (USD); 1 USD = 3.75 SAR] per day as validated by Al-Hada Armed Forces Hospital Bed Management Department. 

​Measures

​The project evaluated outcome and balancing measures to assess the intervention's impact. The primary outcome measures included LOS (measured strictly from admission to discharge), direct financial cost savings calculated from reduced bed-day utilization, and postoperative patient satisfaction scores. Balancing measures safeguarded system equilibrium and patient safety by monitoring complication rates, surgical site infections, and 30-day hospital readmission rates.

​Statistical analysis

Data management and tracking were organized using Microsoft Excel (Microsoft Corporation, Redmond, USA). Run charts were formatted in Canva (Canva Pty Ltd, Sydney, Australia), the driver diagram in Microsoft PowerPoint (Microsoft Corporation, Redmond, USA), and the manuscript and reporting checklist in Word. Statistical analysis was performed using jamovi software (The jamovi project, Sydney, Australia), with all two-tailed tests establishing significance at p < 0.05. Continuous outcomes, such as inpatient LOS, were compared across study phases using the independent Student's t-test. Categorical outcomes, including ambulatory day-case success rates, were evaluated using the Chi-squared (χ^2^ ) test.

## Results

Phase 1: baseline historical cohort (2024)

During the 2024 pre-implementation period, a total of 100 patients scheduled for elective laparoscopic cholecystectomy were evaluated. Following the exclusion of 12 patients according to predefined study criteria, the baseline analytic sample comprised N = 88 patients. Routine surgical care was delivered without a standardized ERAS pathway. All patients were admitted under traditional inpatient protocols, resulting in zero ambulatory day-case discharges (0.0%, n = 0/88). The mean inpatient LOS was 4.33 ± 0.89 days (range: 3.0 to 6.0 days). Systematic tracking of checklist compliance, complications, Surgical site infections, standardized patient satisfaction, and 30-day readmission rates was not established during this baseline phase (Table 1).

**Table 1:** Baseline demographic and clinical characteristics of the historical control cohort (2024) ​Data are presented as n (%) or mean ± SD. N: total population size; n: subgroup sample size; LOS: length of stay; SD: standard deviation.

BASELINE PARAMETER (2024)	SUMMARY VALUE
Total Evaluated Patients	N = 100
Excluded Cases	n = 12 (12.0%)
Final Analytic Sample Size	N = 88 (88.0%)
Ambulatory Day-Case Management (<12h)	0.0% (n = 0/88)
Mean Inpatient LOS	4.33 ± 0.89 days

Phase 2: ERAS implementation & quality improvement cohort (2025)

In 2025, a standardized ERAS clinical pathway was implemented, encompassing 138 evaluated patients. Following 18 exclusions, the active implementation sample comprised N = 120 patients (Table 2).

**Table 2:** Phase 2 ERAS implementation cohort parameters, clinical outcomes, and quality indicators (2025) Data are presented as n (%), mean ± SD, or mean ± SD (range); N: total evaluated population size; n: subgroup sample size; LOS: length of stay; ERAS: enhanced recovery after surgery; SD: standard deviation.

ERAS IMPLEMENTATION PARAMETER (2025)	SUMMARY VALUE
Total Evaluated Patients	N = 138
Excluded Cases	(n = 18 (13.0%
Final Analytic Sample Size	n = 120 (87.0%)
Ambulatory Day-Case Success (<12h)	27.5% (n = 33/120)
Mean Inpatient LOS	2.67 ± 1.15 days
Mean ERAS Protocol Compliance	72.1% ± 38.7% (Range: 0.0%–100.0%)
Mean Patient Satisfaction Score	89.4% ± 3.3% (Range: 85.0%–95.0%)
Overall Complication Rate	0.83% (n = 1/120)
Surgical Site Infections	0.0% (n = 0/120)
30-Day Readmission Rate	0.83% (n = 1/120)

Full compliance was defined as fulfilling ≥ 80% of ERAS perioperative elements per patient. Implementation enabled structured tracking, with monthly compliance increasing from 0.0% (January- February) to a sustained 100.0% from July through December (mean: 72.1% ± 38.7%). Ambulatory day-case success (< 12-hour discharge) reached 27.5% (n = 33/120), while overall mean inpatient LOS decreased to 2.67 ± 1.15 days (range: 1.0-4.0 days) (Figure 3). 

**Figure 3:**
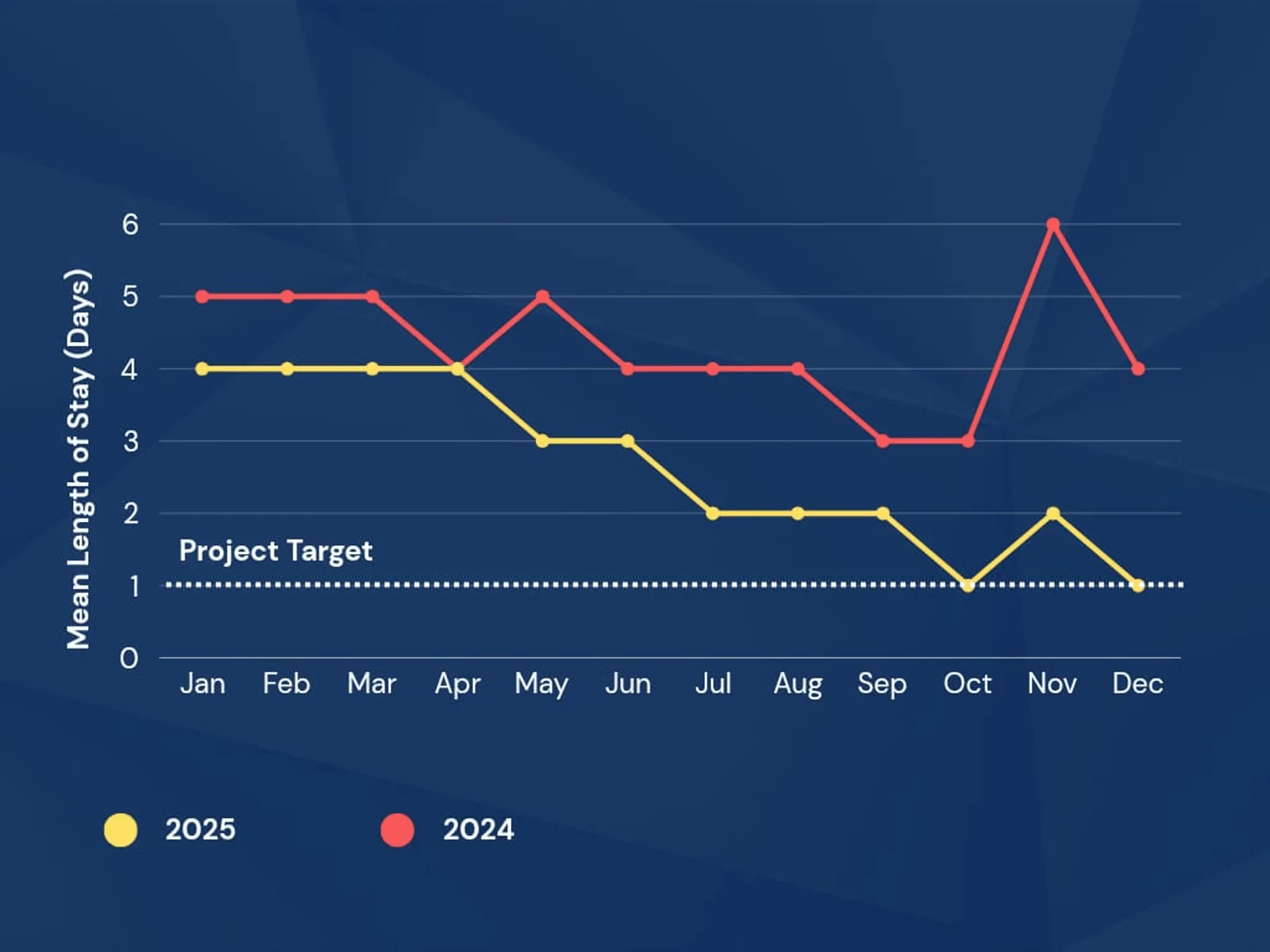
Run chart of monthly mean inpatient LOS before and during ERAS implementation (2024–2025) LOS: length of stay; ​ERAS: enhanced recovery after surgery; PDSA: plan-do-study-act.

Patient satisfaction averaged 89.4% ± 3.3% (peaking at 95.0%), and the 30-day readmission rate was limited to a single patient (0.83%, n = 1/120). Surgical site infections were absent across the entire cohort (0.0%, n = 0/120), and the overall complication rate was 0.83% (n = 1/120), limited to a single patient who developed postoperative jaundice.

Comparative analysis between phases

A direct statistical comparison between the 2024 Baseline and 2025 ERAS Implementation phases demonstrated statistically significant improvements across primary outcome measures:

Key comparative findings

Ambulatory Day-Case Conversion

Day-case management expanded from 0.0% (0/88) in 2024 to 27.5% (33/120) in 2025 (χ² = 26.74, df = 1, p < 0.0001, Cramér's V = 0.36, medium-to-large effect size).

Inpatient LOS Reduction

Average monthly LOS significantly decreased from 4.33 ± 0.89 days in 2024 to 2.67 ± 1.15 days in 2025 (t = 3.96, df = 22, p = 0.0007, Cohen's d = 1.62, very large effect size) (Table 3).

**Table 3:** Comparative analysis of primary clinical outcomes between baseline (2024) and ERAS implementation (2025) phases Data are presented as n (%) or mean ± SD. N: total population size; ERAS: enhanced recovery after surgery; df: degrees of freedom;  χ²: Chi-Square Statistic; V: Cramér’s V; LOS: length of stay; t: Student’s t-Statistic; d: Cohen’s d.

OUTCOME METRIC	2024 BASELINE (N = 88)	2025 ERAS (N = 120)	TEST STATISTIC (DF)	P-VALUE	EFFECT SIZE
Ambulatory Day Case (<12h)	0.0% (0/88)	27.5% (33/120)	χ² = 26.74 (1)	< 0.0001	Cramér's V = 0.36
Mean Inpatient LOS (Days)	4.33 ± 0.89	2.67 ± 1.15	t = 3.96 (22)	0.0007	Cohen's d = 1.62

Economic impact and resource utilization

Implementation of the ERAS protocol generated substantial institutional economic impact. Between May and December 2025, the protocol saved a total of 231 inpatient bed days across 77 surgical patients, achieving total direct hospital cost savings of SAR 346,500 ($92,400 USD) (Table 4). 

**Table 4:** Institutional economic impact and resource utilization following ERAS protocol implementation (May–December 2025) SAR: Saudi Riyal; N: total patient cohort size; LOS: length of stay; USD: United States Dollar; ERAS: enhanced recovery after surgery; MODHS: Ministry of Defense Health Services.

Resource and Economic Parameter	Value
Average Cost of Inpatient Bed Per Day (SAR)	1,500
Total Patient Cohort Managed (N)	77
Mean Inpatient LOS Reduction (Days/Patient)	3
Cumulative Inpatient Bed Days Saved	231 (77 × 3)
Total Projected Financial Savings (SAR)	346,500 (231 × 1,500) ($92,400 USD)

## Discussion

Clinical outcomes and LOS optimization

Optimizing multimodal analgesia, early mobilization, and prompt oral intake enabled our standardized ERAS pathway to reduce mean LOS from four days to one, achieving a 27.5% ambulatory day-case rate with one 30-day readmission, over 90% patient satisfaction, zero surgical site infections, and a single minor complication (postoperative jaundice) over the 12-month project period. Reclaiming 231 cumulative bed-days yielded SAR 346,500 in direct cost savings over eight months, showing that checklist-driven ERAS pathways offer a scalable model to expand surgical throughput and safely relieve hospital capacity constraints. These outcomes align with international evidence demonstrating that ERAS protocols across colorectal, bariatric, and hepatic surgeries significantly reduce hospitalization, complications, and healthcare costs while accelerating recovery [11-14]. Locally, Saudi Arabian data in laparoscopic cholecystectomy and bariatric surgery mirror these broad clinical and satisfaction benefits [12]. Notably, our findings diverge from published literature in two key areas. First, while meta-analyses report a significant increase in 30-day readmissions following upper gastrointestinal (GI) surgeries [14], our cohort had one readmission. Second, while ERAS benefits are well established in complex hepatic, cystectomy, and colorectal procedures [11,13], our project shows that structured implementation can safely convert a high-volume routine procedure like laparoscopic cholecystectomy into an ambulatory surgery. Ultimately, perioperative standardization serves as a high-value operational strategy for healthcare facilities seeking to optimize resource utilization and expand elective surgical capacity. 

Process quality indicator auditing and localized data gaps

Our localized approach directly echoes established evidence that continuous tracking of process quality indicators is essential to uncover practice gaps and overcome implementation barriers. While traditional centralized systems often rely on expansive, resource-intensive frameworks, localized monitoring offers an efficient alternative for continuous clinical audit [15]. Over the 12-month implementation phase, protocol compliance rose from 0.0% at baseline to a sustained 100.0% from July through December (mean compliance: 72.1% ± 38.7%). Systematically tracking how these protocols were operationalized provided actionable insights to eliminate compliance barriers in real time. While regional data in Saudi Arabia has primarily validated ERAS pathways in other high-volume procedures, such as sleeve gastrectomy in Jeddah, a localized data gap remained regarding gallbladder surgeries [16]. By proving that a checklist- and leadership-driven model can safely expand day-case management, this project bridges that gap, offering a reproducible framework to maximize resource efficiency.

Implementation innovation, sustainability, and hospital-wide scaling 

While literature establishes that ERAS pathways significantly optimize recovery after laparoscopic cholecystectomy [17], this initiative transitions that paradigm into a sustainable institutional reality. Moving beyond passive guideline adoption, the primary innovation of this framework lies in its low-resource implementation strategy: utilizing cross-disciplinary leadership and a low-cost, file-integrated checklist that operates efficiently without requiring expensive digital infrastructure. Long-term sustainability is supported and maintained by embedding this checklist into formal hospital standard operating procedures (SOPs), backed by regular automated electronic health record (EHR) compliance audits and oversight from an empowered ERAS Steering Committee. Crucially, following the demonstrated clinical success and operational feasibility in laparoscopic cholecystectomy, this initiative has been scaled across all surgical subspecialties within our institution, incorporating tailored ERAS checklists directly into the EHR for all surgical patients. Because this expanded framework leverages existing hospital infrastructure and routine local feedback, it provides a highly scalable, self-sustaining blueprint adaptable to diverse surgical disciplines across the region.

Limitations

Staff Engagement and Protocol Adherence Barriers

Initial traditional education failed to reduce LOS. However, transitioning to a mandatory, file-integrated ERAS checklist driven by active joint leadership from anesthesia and general surgery closed the baseline engagement gap, achieving 100% protocol compliance and a subsequent decline in LOS.

Infrastructure Limitations and Operating Hours

Second, institutional infrastructure and administrative schedules created structural barriers to optimizing ambulatory care outcomes within the day surgery unit at Alhada Armed Forces Hospital, which currently operates only until 5:00 PM. Consequently, patients undergoing laparoscopic cholecystectomy after 12:00 PM could not achieve the required post-operative monitoring window before unit closure, necessitating standard inpatient admission from the outset. This operational cutoff artificially capped the volume of true day-case tracking at 27.5%. Expanding the day surgery unit's operating hours until 12:00 AM represents a strategic institutional opportunity to shift the vast majority of late-afternoon surgical cohorts into true ambulatory pathways, further maximizing resource efficiency. 

## Conclusions

Integrating a mandatory, file-embedded ERAS checklist under multidisciplinary leadership successfully standardizes laparoscopic cholecystectomy care. This low-resource initiative compresses LOS, drives institutional cost savings, and expands day-case discharge without compromising safety or satisfaction. Grounded in robust patient education and active engagement, this adaptable model offers a practical framework for regional health systems, where extended day-surgery hours serve as a straightforward way to increase hospital capacity.
